# Establishment of stable iPS-derived human neural stem cell lines suitable for cell therapies

**DOI:** 10.1038/s41419-018-0990-2

**Published:** 2018-09-17

**Authors:** Jessica Rosati, Daniela Ferrari, Filomena Altieri, Silvia Tardivo, Claudia Ricciolini, Caterina Fusilli, Cristina Zalfa, Daniela C. Profico, Francesca Pinos, Laura Bernardini, Barbara Torres, Isabella Manni, Giulia Piaggio, Elena Binda, Massimiliano Copetti, Giuseppe Lamorte, Tommaso Mazza, Massimo Carella, Maurizio Gelati, Enza Maria Valente, Antonio Simeone, Angelo L. Vescovi

**Affiliations:** 1 Cellular Reprogramming Unit, IRCCS Casa Sollievo della Sofferenza, Viale dei Cappuccini, 71013 San Giovanni Rotondo, Foggia Italy; 2 Department of Biotechnology and Biosciences, University of Milan Bicocca, Piazza della Scienza, 220126 Milan, Italy; 3 Neurogenetics Unit, IRCCS Santa Lucia Foundation, Rome, Italy; 4 Stem Cell Laboratory, Cell Factory e Biobank, Terni Hospital, Via Tristano di Joannuccio 1, 05100 Terni, Italy; 5 Bioinformatics Unit, IRCCS Casa Sollievo della Sofferenza, Viale dei Cappuccini, 71013 San Giovanni Rotondo, Foggia Italy; 6 Production Unit of Advanced Therapies (UPTA), Institute for Stem-Cell Biology, Regenerative Medicine and Innovative Therapies (ISBReMIT), IRCCS Casa Sollievo della Sofferenza, Viale dei Cappuccini, 71013 San Giovanni Rotondo, Foggia Italy; 7 Medical Genetics Unit, IRCCS Casa Sollievo della Sofferenza, Viale dei Cappuccini, 71013 San Giovanni Rotondo, Foggia Italy; 8 Department of Research, Diagnosis and Innovative Technologies, Regina Elena National Cancer Institute, Rome, Italy; 9 Cancer Stem Cells Unit (ICS), Institute for Stem-Cell Biology, Regenerative Medicine and Innovative Therapies (ISBReMIT), IRCCS Casa Sollievo della Sofferenza, Viale dei Cappuccini, 71013 San Giovanni Rotondo, Foggia Italy; 10 Biostatistic Unit, IRCCS Casa Sollievo della Sofferenza, Viale dei Cappuccini, 71013 San Giovanni Rotondo, Foggia Italy; 11 Department of Molecular Medicine, University of Pavia, Via Forlanini 14, 27100 Pavia, Italy; 12 Institute of Genetics and Biophysics Adriano Buzzati Traverso, CNR, Via P. Castellino 111, 80131 Naples, Italy; 13 IRCSS Neuromed, 86077 Pozzilli, Isernia Italy

## Abstract

Establishing specific cell lineages from human induced pluripotent stem cells (hiPSCs) is vital for cell therapy approaches in regenerative medicine, particularly for neurodegenerative disorders. While neural precursors have been induced from hiPSCs, the establishment of hiPSC-derived human neural stem cells (hiNSCs), with characteristics that match foetal hNSCs and abide by cGMP standards, thus allowing clinical applications, has not been described. We generated hiNSCs by a virus-free technique, whose properties recapitulate those of the clinical-grade hNSCs successfully used in an Amyotrophic Lateral Sclerosis (ALS) phase I clinical trial. Ex vivo, hiNSCs critically depend on exogenous mitogens for stable self-renewal and amplification and spontaneously differentiate into astrocytes, oligodendrocytes and neurons upon their removal. In the brain of immunodeficient mice, hiNSCs engraft and differentiate into neurons and glia, without tumour formation. These findings now warrant the establishment of clinical-grade, autologous and continuous hiNSC lines for clinical trials in neurological diseases such as Huntington’s, Parkinson’s and Alzheimer’s, among others.

## Introduction

Cell therapy remains one of the most promising approaches for the treatment of neurological disorders. Recent observations of improved motor function in Parkinson’s patients as elicited from transplanted mesencephalic dopaminergic neurons, suggest that the harnessing of the healing potential of these techniques may finally be within our reach^1^. However, many of the currently accessible cell systems present us with serious hurdles, pertaining to donor tissue procurement, heterogeneity, availability and related technical or ethical concerns^2–5^.

Many of these issues could be alleviated by the use of stem cells, whose inherent expansion ability and functional plasticity could respectively increase availability and trigger therapeutic actions, such as the replacement of dead cells, immunomodulation, anti-inflammatory, trophic and homeostatic activities^6–13^. For a systematic clinical use of neural stem cells (NSCs)^14–18^, manipulation systems and preparations must guarantee the broad availability of donor cells with reproducible cell behaviour and therapeutic effects through (1) expression of the full complement of stem cell functional characteristics and (2) stable and extensive self-renewal properties.

We have recently stated that stable human NSCs (hNSCs) can satisfy these requirements. Having obtained current good manufacturing practices (cGMP) certification for hNSCs from miscarriages, we have successfully used them in a phase I trial, with intraspinal transplantation in 18 ALS patients^15^. We are now focusing on resolving the concerns deriving from the use of allogeneic hNSCs and related immune suppression^19^. Since the establishment of autologous hNSCs is both impractical and, de facto, impossible, we have derived these cells from autologous human induced pluripotent stem cells (hiPSCs).

Recently, various types of central nervous system (CNS) precursors have been derived from hiPSCs^20–22^; nonetheless, evidence of systems for establishing bona fide, hiPSC-derived hNSCs endowed with the complete range of defining stem cell characteristics is negligible^20^. We describe a reproducible system to establish stable hiNSCs, whose properties recapitulate those of hNSCs. This takes place under conditions that avoid foreign DNA integration and that should allow for certification of the emerging hiNSCs according to cGMP guidelines and their potential use for autologous cell therapy.

## Results

### Generation and characterisation of hiPSCs

**Fig. 1:**
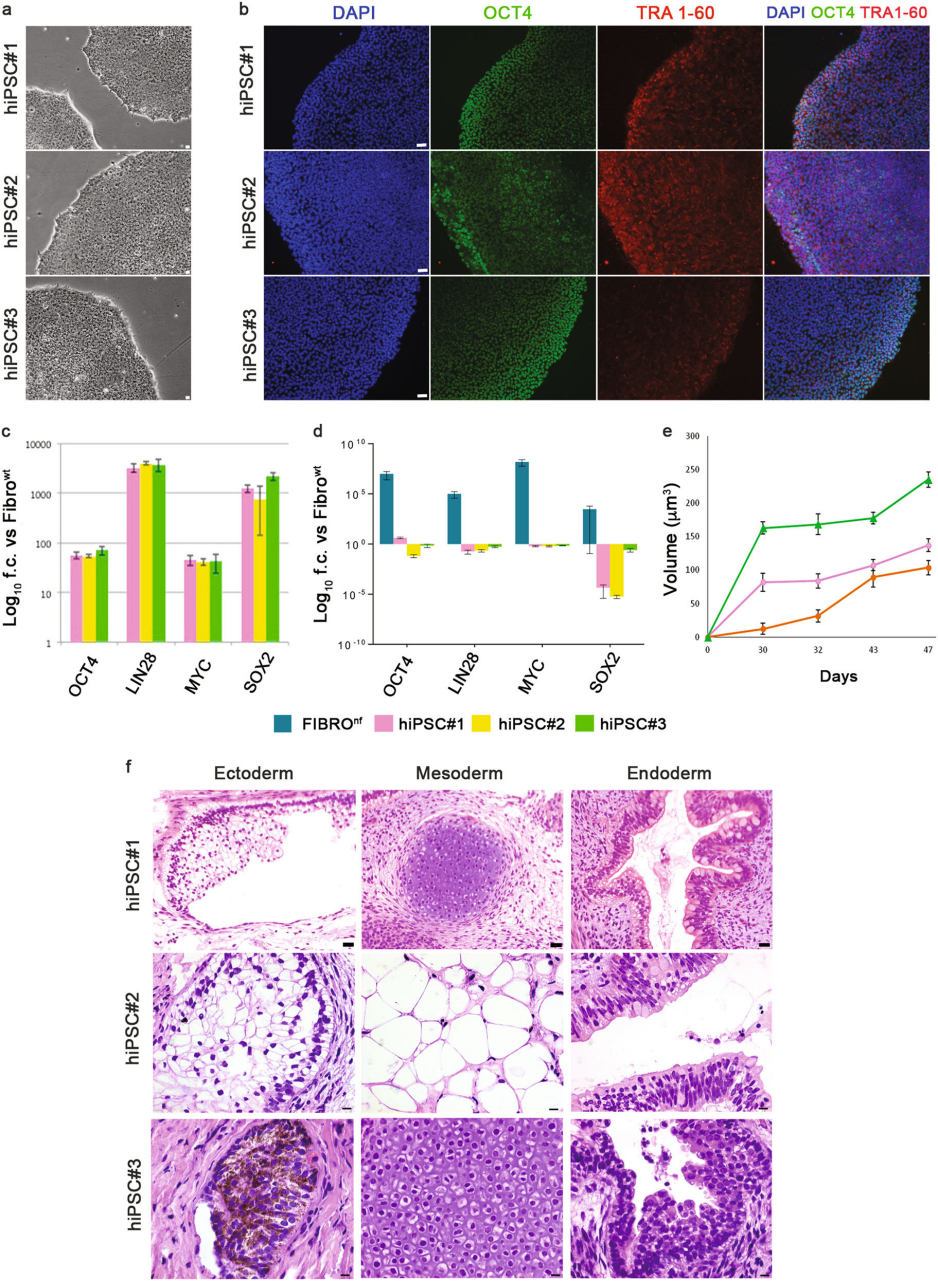
Characterisation of adult skin fibroblast-derived hiPSC lines. **a** Phase contrast of hiPSC#1, hiPSC#2 and hiPSC#3. **b** Immunofluorescence images showing expression of OCT4 (green) and TRA-1-60 (red) in hiPSCs lines. Nuclei are counterstained with DAPI (blue). **c** Histograms showing mRNA expression of *OCT4*, *LIN28*, *L-MYC* and *SOX2* in hiPSCs with respect to non-nucleofected, wild-type fibroblasts (Fibro^wt^). Data are normalised on actin expression, are shown as log_10_ fold changes (f.c.) and represent the mean ± SEM of three experiments in duplicate. **d** Histograms showing the absence of exogenous genes expression after five–six passages of hiPSC amplification. Nucleofected fibroblasts (Fibro^nf^) are used as positive control. Data are normalised on actin expression, are shown as log_10_ fold changes (f.c.) and represent the mean ± SEM of three experiments in duplicate. **e** Growth curves of hiPSC-derived teratomas. **f** Histological analysis of teratomas generated by hiPSCs after subcutaneous injection in immunodeficient mice. Representative hematoxylin-eosin images showing the presence of ectodermal derivatives (sebaceous gland for hiPSC#1 and hiPSC#2, neuroepithelial rosette with evident melanin deposits for hiPSC#3), mesodermal derivatives (cartilage for hiPSC#1 and hiPSC#3, adipose tissue for hiPSC#2) and endodermal derivatives (intestinal epithelium for hiPSC#1 and non-keratinised epithelial lining for hiPSC#2 and hiPSC#3). Scale bars: in **a** and **b** = 50 μm, in **f** = 20 μm for hiPSC#1 and 10 μm for hiPSC#2 and hiPSC#3

We generated virus-free hiPSCs from human skin fibroblasts using a non-integrating, episomal-based reprogramming system, under feeder-free and xeno-free conditions suitable for obtaining cGMP certification^23–25^. Data are from three distinct lines: hiPSC#1, hiPSC#2 and hiPSC#3, from healthy, consenting adults^26^. hiPSCs displayed a typical human embryonic stem cell (hESC) morphology (Fig. 1a) and expressed OCT4 and TRA-1-60 (Fig. 1b and Suppl. Figure 1a). The endogenous expression (Fig. 1c), and the absence of exogenous expression (Fig. 1d) of the pluripotency markers LIN28, OCT4, KLF4, SOX2 and L-MYC were demonstrated through quantitative real-time PCR (qRT-PCR). As expected, hiPSC#1, hiPSC#2 and hiPSC#3 produced teratomas upon subcutaneous injection in immunodeficient mice (Fig. 1e, f and Suppl. Figure 1b–e). The karyotype of each hiPSC line (46, XX) was normal (>20 passages, Suppl. Figure 2a). Only one (out of three cellular lines) contained a minor copy number variation (CNV) produced by cell amplification, maintained in the neurospheres without further genome modifications^65,64,64^. hiPSCs were mycoplasma-free (Suppl. Figure 2b). Thus, these lines fulfilled criteria for identifying properly reprogrammed hiPSCs.

### Generation of hiNSCs

**Fig. 2:**
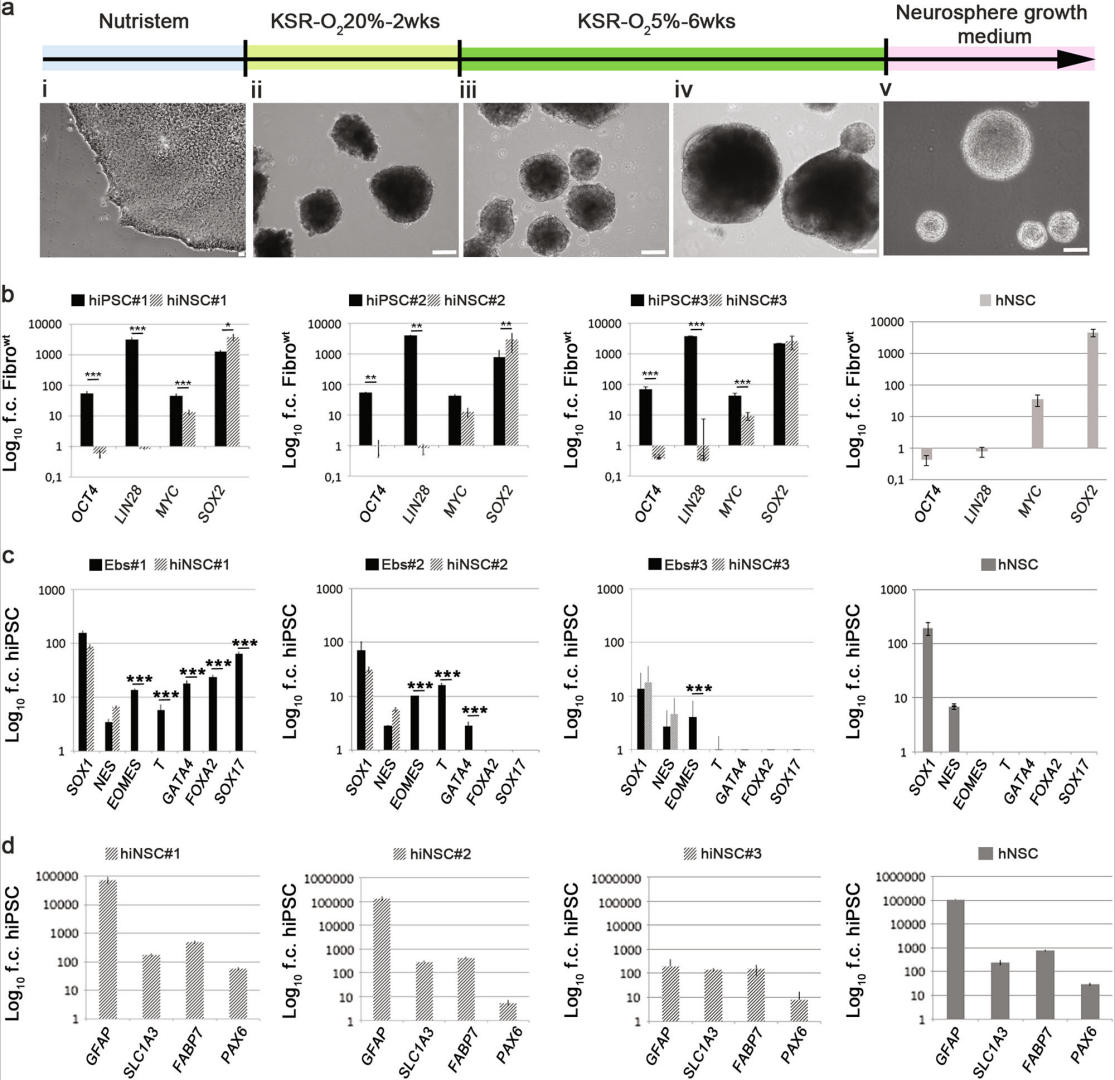
Generation and characterisation of hiNSCs. **a** Schematic diagram illustrating the overall strategy to generate hiNSCs from hiPSCs. Phase contrast images illustrate the typical morphology of cells at each stage. **b** Bar graphs of qRT-PCR showing mRNA-expression profile of hiNSCs, hiPSCs and brain-derived hNSCs respect to Fibro^wt^. **c** Bar graphs of qRT-PCR showing mRNA-expression profile of EBs, hiNSCs and hNSCs respect to hiPSCs. **d** Bar graphs of qRT-PCR showing mRNA-expression profile of hiNSCs and hNSCs respect to hiPSCs. Each bar represents the mean ± SEM of three independent experiments in duplicate. Data are expressed as log_10_ fold changes (f.c.) respect to non-nucleofected fibroblasts (**b**, Fibro^wt^) or hiPSCs (**c**, **d**). Data are normalised on actin expression. Scale bars: **a**, i = 50 μm; **a**, ii–v = 100 μm

We hypothesised that NSCs would likely appear throughout embryoid body (EB) differentiation and that by applying the selective culture conditions favouring the growth of hNSCs, stem cells might be isolated and expanded at the expense of other cell lineages, as shown with primary CNS tissue^8,27^. To optimise the protocol and timing of hiNSC generation and amplification, we tracked the expression of radial glial markers throughout differentiation of EBs. We used BLBP^28,29^, GLAST^30,31^, GFAP^32^ and PAX6^33^ as markers of the neuralisation onset, for up to 8 weeks after triggering EB formation. Small subsets of EB cells acquired radial glial characteristics beginning as early as 2 weeks from EB induction (not shown). Yet, at this stage, efficient expansion of hiNSCs failed, most likely due to the small population size. Therefore, EBs were grown in KnockOut Serum Replacement (KSR) medium for 2 weeks under normoxia (Fig. 2a, i, ii) and switched to hypoxic conditions for 6 additional weeks (Fig. 2a, iii, iv). Several translucent, lightly stained protrusions emerging from dark EBs were observed (Fig. 2a, iv). After 8 weeks, KSR was replaced with neurosphere growth medium, routinely used to establish continuous, clinical-grade hNSCs from miscarriages^8,15^. EBs, mechanically dissociated after 10 days, produced round clusters similar to neurospheres, which were serially amplified every 10–15 days (Fig. 2a, v), giving rise to functionally stable, steadily expanding lines. The lines were banked through cryopreservation, displaying 60–70% vitality upon thawing. Karyotypes remained stable, mycoplasma absent (Suppl. Figure 3). The bona fide nature of the hiNSCs was assessed molecularly and functionally in comparison to GMP-grade hNSC produced according to European Medicines Agency standards (AIFA aM 54/2018) and used in phase I trials for ALS patients (NCT01640067) and for secondary progressive Multiple Sclerosis (MS) patients (NCT03282760).

### Molecular characterisation of hiNSCs

Our first step was to exclude the permanence of cells expressing hiPSC markers in our hiNSC cultures. Expression of hiPSC markers in hiNSC#1, hiNSC#2 and hiNSC#3 was compared to their parental hiPSCs and to hNSCs (Fig. 2b). Differently from hiPSCs, neither hiNSCs nor hNSCs expressed detectable levels of pluripotency markers, such as OCT4 and LIN28. Conversely, L-MYC and NSC-putative marker SOX2 were expressed by both hiNSCs and hNSCs.

To exclude the presence of non-neuro-ectodermal cells, we compared the expression of mesodermal, endodermal and neuroectodermal lineage markers of the three hiNSC lines to EBs and hNSCs. hiNSCs did not express EOMES, T, GATA4, FOXA2 and SOX17, but retained a high expression of the early neural antigens SOX1 and NES, similar to hNSCs (Fig. 2c). Radial glial markers were consistently expressed in both hiNSCs and hNSCs throughout serial subculturing, confirming their similarity (Fig. 2d).

In conclusion, our hiNSCs were selectively positive only for neuro-ectodermal markers. These results suggest that the application of the neurosphere selection method enriched/amplified exclusively neural progenitors possessing hNSC molecular characteristics.

### Functional in vitro and in vivo characterisation and safe expansion of hiNSCs

We then compared our hiNSCs to clinical-grade hNSCs using the neurosphere assay^27,34–36^, in order to verify whether our hiNSCs possessed the ex vivo functional characteristics of bona fide NSCs—extensive self-renewal and multipotency in particular—that are epitomised in native hNSCs^8,37^.

#### Stem cell properties of hiNSCs

When plated under the stringent conditions of the neurosphere assay, hNSCs form the typical neurospheres that we have also consistently observed in our hiNSC cultures (Fig. 2a, v). Moreover, if the perpetuation of the culture is factually sustained by true hNSCs^8,15^, hNSCs expand to large numbers, stably retaining their growth curve slopes over extensive passages^8^.

**Fig. 3:**
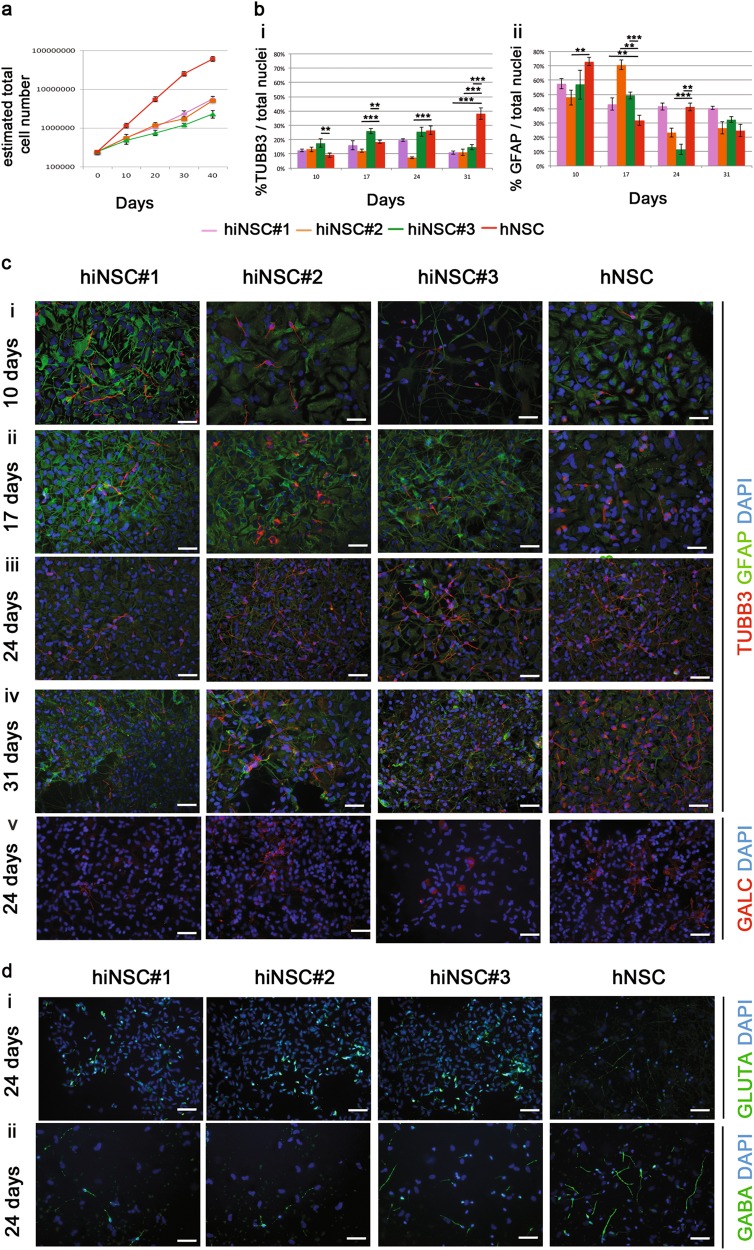
Stem cell properties of hiNSCs. **a** Growth curves of hiNSC lines (passages 10–15) compared to brain-derived hNSCs in neurosphere growth medium. Each value point of the curve is the mean ± SEM of three independent experiments. **b** Histograms showing the quantification of i TUBB3 + neurons and ii GFAP + astrocytes out of total nuclei, after in vitro differentiation of hiNSC and hNSC lines. Each bar represents the value of three independent experiments ± SEM. Only the statistical differences between each single hiNSC line with respect to the hNSCs are shown. **c** Representative fluorescent images showing hiNSC-derived neurons (TUBB3, red, i–iv), astrocytes (GFAP, green, i–iv) and oligodendrocytes (GALC, red, v) at days i 10, ii 17, iii 24 and iv 31 upon differentiation. **d** Expression of i GLUTA (green) and ii GABA (green) in hiNSC and brain-derived hNSC progeny, after 24 days of in vitro differentiation. Scale bars: 50 μm

All three hiNSCs displayed this behaviour, though at a lower expansion rate than hNSCs (twofold versus fivefold every 10–15 days for over 15 passages (Fig. 3a). Starting from as low as 250 000 hiNSCs, the estimated cell number that can be generated exceeds 4 × 10^9^, showing that hiNSCs possess the expected extensive self-renewal properties that also make them appropriate for cGMP certification. The protocol was then tested on additional hiNSCs from healthy and affected individuals^38–40^, demonstrating similar growth curves to hNSCs, with minimum variabilities due to genetic background.

We then assessed if our hiNSCs displayed multipotency, i.e. the ability to differentiate into cells of the three main CNS lineages. hiNSCs and hNSCs were differentiated in parallel and detection of neuronal class III β-tubulinIII (TUBB3; neurons), glial fibrillary acidic protein (GFAP; astroglia) and GALC (oligodendroglia) was analysed at 10, 17, 24 and 31 days in vitro (DIV) (Fig. 3b and Suppl. Figure 4a).

At 10 DIV, immature neurons were detected, which matured within 17 DIV, generating a dense network of long-branched TUBB3-positive processes with varicosities up to 31 DIV (Fig. 3c, i–iv and Suppl. Figure 4b), which also expressed the late-stage neuronal marker MAP2 (Suppl. Figure 4c). We further observed progressive morphological changes during GFAP-positive astrocyte differentiation (Fig. 3c, i–iv and Suppl. Figure 4b). GALC-positive oligodendrocytes were detected at all time intervals for all hiNSCs (Fig. 3c, v and Suppl. Figure 5a).

The quantitative analysis (Fig. 3b, i, ii) showed comparable patterns of differentiation for all hiNSCs. At 10 DIV the percentage of TUBB3+ was 12.4% ± 1%, 13.18% ± 1.7% and 17.60% ± 3.03% for hiNSC#1, #2 and #3, respectively, and remained generally stable for up to 31 DIV (10.88% ± 1.1%, 11.23% ± 2.3% and 14.66% ± 1.8% for hiNSC#1, #2 and #3). The percentages of GFAP+ at 10 DIV were also comparable for hiNSCs (57.62% ± 3.6%, 47.86% ± 5.3% and 56.82% ± 10.10%, for hiNSC#1, #2 and #3) and slowly decreased over time (40.5% ± 1.1%, 26.58% ± 4.2% and 32.46% ± 2.2% at 31 DIV for hiNSC#1, #2 and #3). The number of GALC-positive cells was stable for up to 31 days (8.53% ± 0.78%, 12.39 ± 1.33%, 6.56 ± 2.9% for hiNSC#1, #2 and #3) (Fig. 3c, v and Suppl. Figure 5a). Considering that the total neural cell percentage at 31 DIV was circa 60%, we stained for NESTIN, confirming that a part of the population had not yet managed to progress in its differentiation (Suppl. Figure 5b).

The qualitative differentiation profiles of hNSCs and hiNSCs were similar, although quantitative analyses showed significant differences, (Fig. 3b, i, ii).

An initial analysis of the neurotransmitter phenotypes generated by hiNSCs showed that as seen previously for hNSCs^8^, a small fraction of these were partly glutamatergic (4.90% ± 0.8%, 6.03% ± 2.1% and 1.98% ± 0.3% for hiNSC#1, #2 and #3, GLUTA+, Fig. 3d, i and Suppl. Figure 4e) and partly gabaergic (7.92% ± 1.32%, 8.83% ± 1.8% and 7.13% **±** 1.0% for hiNSC#1, #2 and #3, GABA+, Fig. 3d, ii and Suppl. Figure 4d).

### Safety considerations: evaluation of proliferation activity

**Fig. 4:**
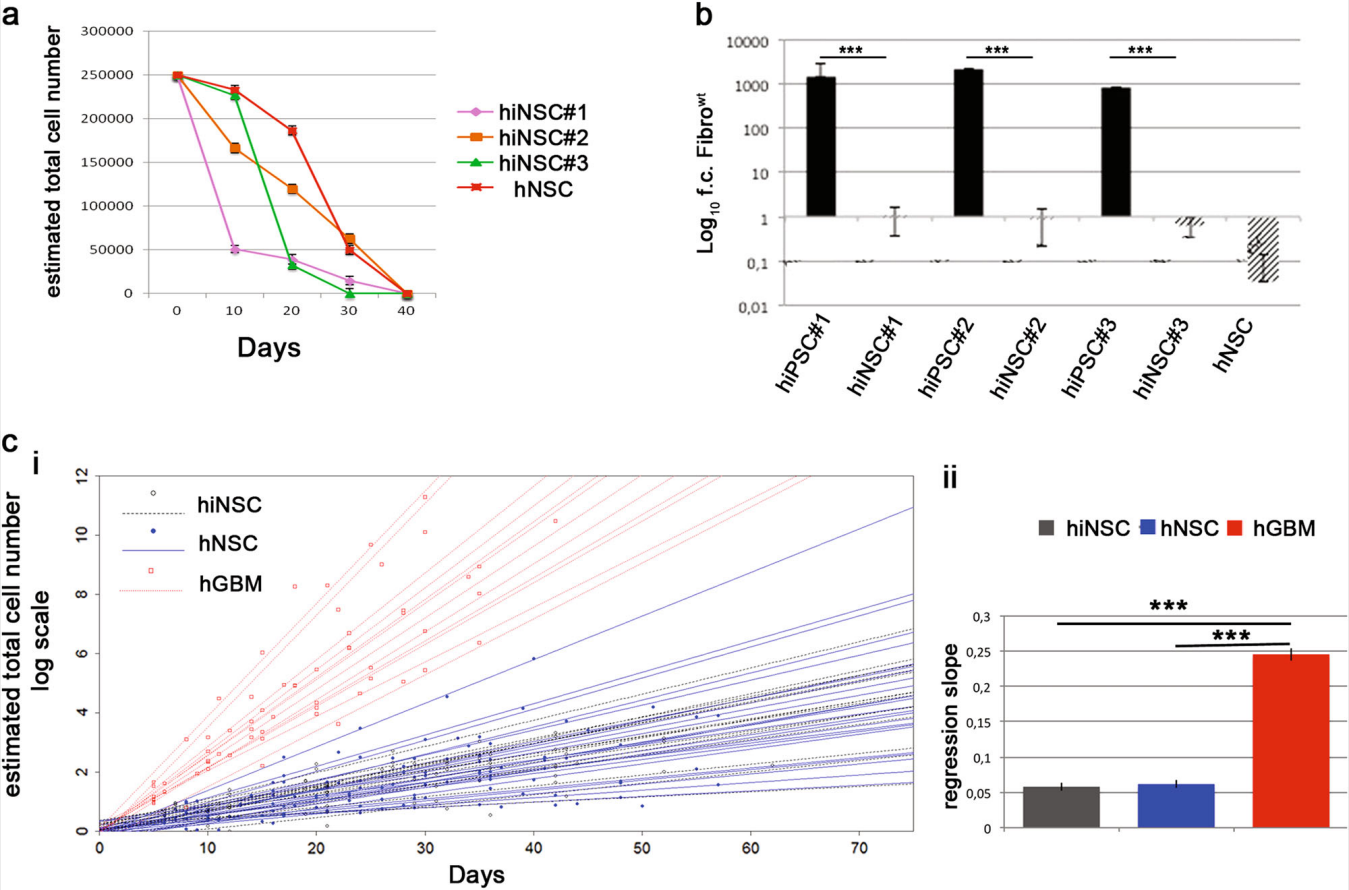
Safe expansion of hiNSC lines. **a** Growth factor-deprivation curves demonstrating the growth factors dependence of hiNSC lines. **b** qRT-PCR showing downregulation of *hTERT* mRNA expression in hiNSCs respect to hiPSCs. Each bar represents the mean ± SEM of three independent experiments for each clone (hiPSCs and hiNSCs) in duplicate. Data are expressed as log_10_ fold changes (f.c.) respect to non-nucleofected fibroblasts (Fibro^wt^) and are normalised on actin expression. **c** Comparison between the growth curves (i) and their slope (ii) of hiNSC (*n* = 15), respect to brain-derived hNSCs (*n* = 32) and hGBM (*n* = 12)

To exclude the possibility that hiNSCs might acquire uncontrolled growth capacity, prodromal to cell transformation and tumorigenicity, we verified that hiNSC retained a permanent cellular mitogen dependence^15^, a key functional test for cGMP hNSCs. When hiNSCs were cultured without growth factors, a progressive loss of proliferation capacity was observed, with the number of viable cells dropping to zero in five passages or fewer, as in hNSCs (Fig. 4a).

We evaluated *hTERT* expression, a hallmark of transformed brain stem cells^41^, absent in hNSCs. We found no expression of *hTERT* in the hiPSC-parental fibroblasts and, as expected, a high expression in hiPSCs, as a consequence of their successful reprogramming (Fig. 4b). Notably, telomerase expression became undetectable in hiPSC-derived hiNSCs, as in the hNSCs used as external controls (Fig. 4b).

These findings were confirmed through a statistical analysis comparing the kinetic growth slopes of three cell groups: (1) hiPSC-derived hiNSCs, *n* = 15; (2) hNSCs, used in a clinical phase I trial, *n* = 32; and (3) cancer stem cell lines derived from human glioblastoma (hGBM) specimens^41^, *n* = 12. hiNSCs showed slopes similar to hNSCs (*p* = 0.815) while, as expected, their growth was slower than hGBM cell lines (*p* < 0.0001) (Fig. 4c, i, ii).

### Evaluation of engraftment and lack of tumorigenic ability of hiNSCs in immunodeficient mice

The hiNSC growth characteristics observed above support future applications in clinical settings. hiNSCs were implanted into immunodeficient athymic mice striatum (300 000 cells/animal, *n* = 11) and their growth was compared to animals receiving the same number of cGMP hNSCs (*n* = 10) (negative controls) and hGBM cells (*n* = 3, 150 000 cells/animal) (positive controls).

**Fig. 5:**
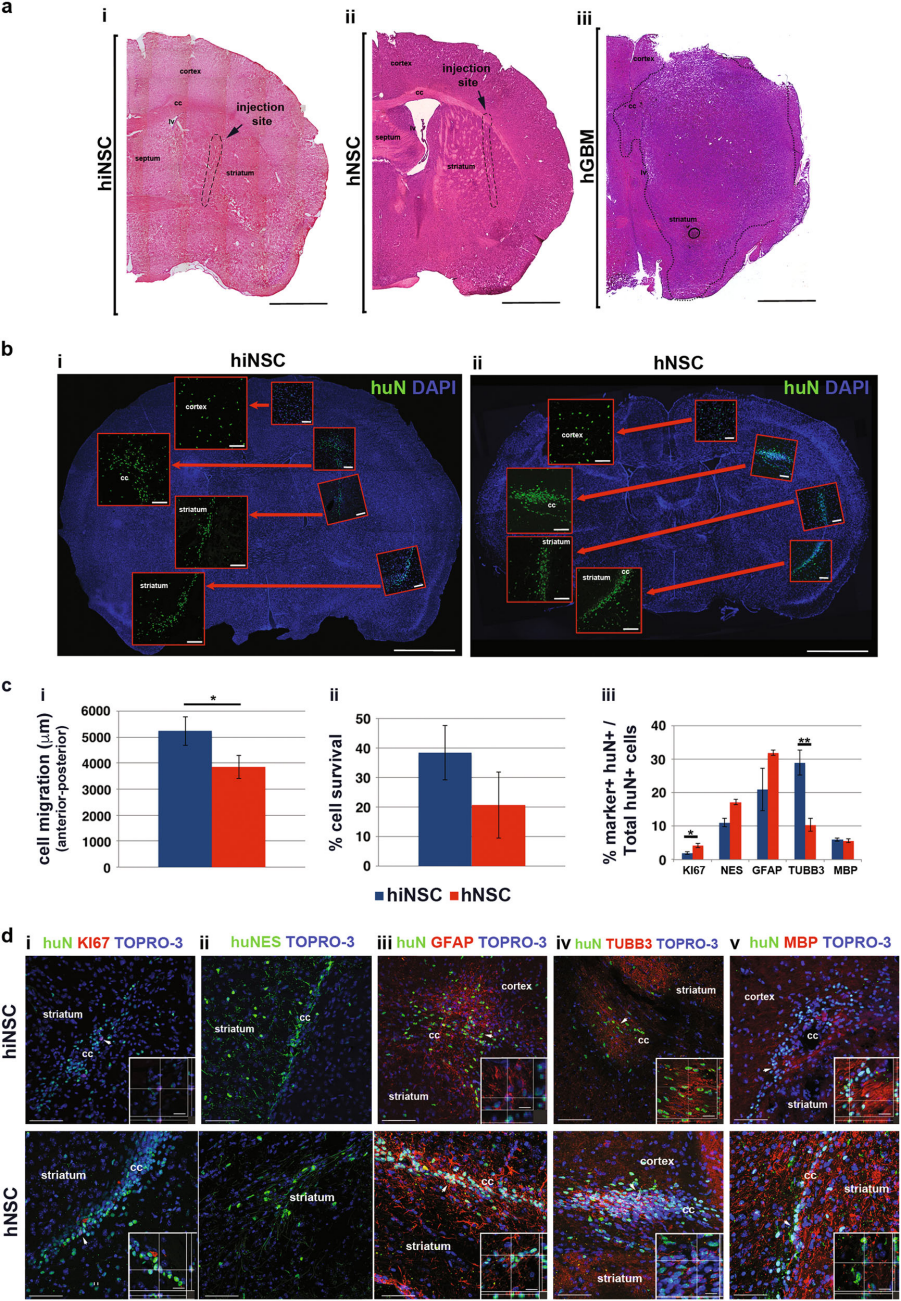
Engraftment and non-tumorigenicity of hiNSCs upon transplantation into the brain. **a** Haematoxilyn-eosin stain of ipsilateral hemisphere of animals transplanted with i hiNCSs (*n* = 11), ii hNSCs (*n* = 10) and iii hGBM cells (*n* = 3). **b** Brain map showing the distribution of transplanted i hiNSCs and ii hNSCs (huN+, green) throughout the brain. In the magnifications are shown the brain regions evaluated for quantification analysis: cortex, striatum, corpus callosum, subventricular zone (not shown) and injection site. **c** Quantification of i migration, ii survival ability and iii of the relative percentages of neurons, astrocytes and oligodendrocytes generated by hiNSCs and hNSCs. **d** Confocal images showing the expression of i proliferation (KI67, red), ii neural (huNES, green), iii astroglial (GFAP, red), iv neuronal (TUBB3, red) and v oligodendroglial (MBP, red) markers for hiNSCs and hNSCs (huN+, green). Nuclei are shown by TO-PRO-3 staining (blue). Scale bar: **a**, **b** = 1000 µm, **d** = 75 µm, **d**, i, ii, v inserts = 17–19 µm, **d**, iv insert = 10 µm

For the entire duration of the study (6 months), the animals receiving either hiNSCs or hNSCs did not present with clinical symptoms emerging from intraparenchymal cell overgrowth, i.e. behavioural anomalies, hydrocephalia, weight loss or ataxia. Brain haematoxilyn/eosin staining showed a normal cytoarchitecture of the striatal parenchyma even in the proximity of the injection site (needle track, Fig. 5a, i, ii) and confirmed the lack of exacerbated proliferation, as opposed to the large tumoral masses observed in mice receiving hGBM (Fig. 5a, iii). Furthermore, the immunohistochemistry showed that hiNSCs successfully engrafted, as shown by human-specific nuclei (huN) expression, migrating from the injection site towards the corpus callosum and cortical regions (Fig. 5b, i), similarly to hNSC behaviour (Fig. 5b, ii). Quantitative analysis of cell migration and survival (Fig. 5c, i, ii) also confirmed similar engraftment abilities between hiNSCs and hNSCs. We observed that hiNSCs seemed to be more prone to migrate from the injection site when compared to hNSCs (antero-posterior graft extension: 5.23 ± 0.55 mm for hiNSCs and 3.84 ± 0.44 mm for hNSCs, *p* < 0.05 (Fig. 5c, i). The total number of viable engrafted cells was 38.39 ± 9.29% for hiNPSCs and 20.68 ± 11.29% for hNSCs, (Fig. 5c, ii). Of note, the percentage of Ki67+ in hiNSCs (1.56 ± 0.44%) was lower than in hNSCs (4.21 ± 0.63%, *p* < 0.05) (Fig. 5c, iii; d, i).

### Analysis of the differentiation pattern of transplanted hiNSCs

We evaluated the ability of hiNSCs to give rise to mature CNS lineages 6 months after intracerebral transplantation. Confocal analysis (Fig. 5d and Suppl Fig. 6) detected huN co-localisation with markers expressed by immature CNS progenitors (NES), astrocytes (GFAP), neurons (Doublecortin (DCX) and TUBB3) and oligodendrocytes (myelin basic protein; MBP), confirming the multilineage differentiation capacity of our hiNSCs, again similar to that of hNSCs. Quantitative analysis (Fig. 5c, iii) was performed on brain slices in the proximity of the injection site, showing that a similar percentage of hiNSCs and hNSCs expressed NES (11.21 ± 1.25% for hiNSCs and 17.18 ± 0.87% for hNSCs, Fig. 5c, iii; d, ii) and GFAP, (21.22 ± 6.37% for hiNSCs and 31.93 ± 0.79% for hNSC, Fig. 5c, iii; d, iii and Suppl. Figure 6). Interestingly, hiNSCs were more prone to differentiate into the neuronal lineage than hNSCs and this feature was evident when comparing both the most immature fraction of huN+ neurons, i.e. migrating neuroblasts positive for DCX+ (19.46 ± 2.93% for hiNSCs and 11.26 ± 0.87% for hNSCs, *p* < 0.05, not shown) and the more mature TUBB3+ neurons (28.82 ± 3.77% for hiNSCs and 10.34 ± 1.89% for hNSCs, *p* < 0.01, Fig. 5c, iii; d, iv and Suppl. Figure 6). Finally, oligodendroglial huN+/MBP+ cells (Fig. 5d, v) were detected in similar percentages: 5.80 ± 0.56% for hiNSCs and 5.60 ± 0.60% for hNSCs (Fig. 5c, iii and Suppl. Figure 6).

### Gene expression analysis

**Fig. 6:**
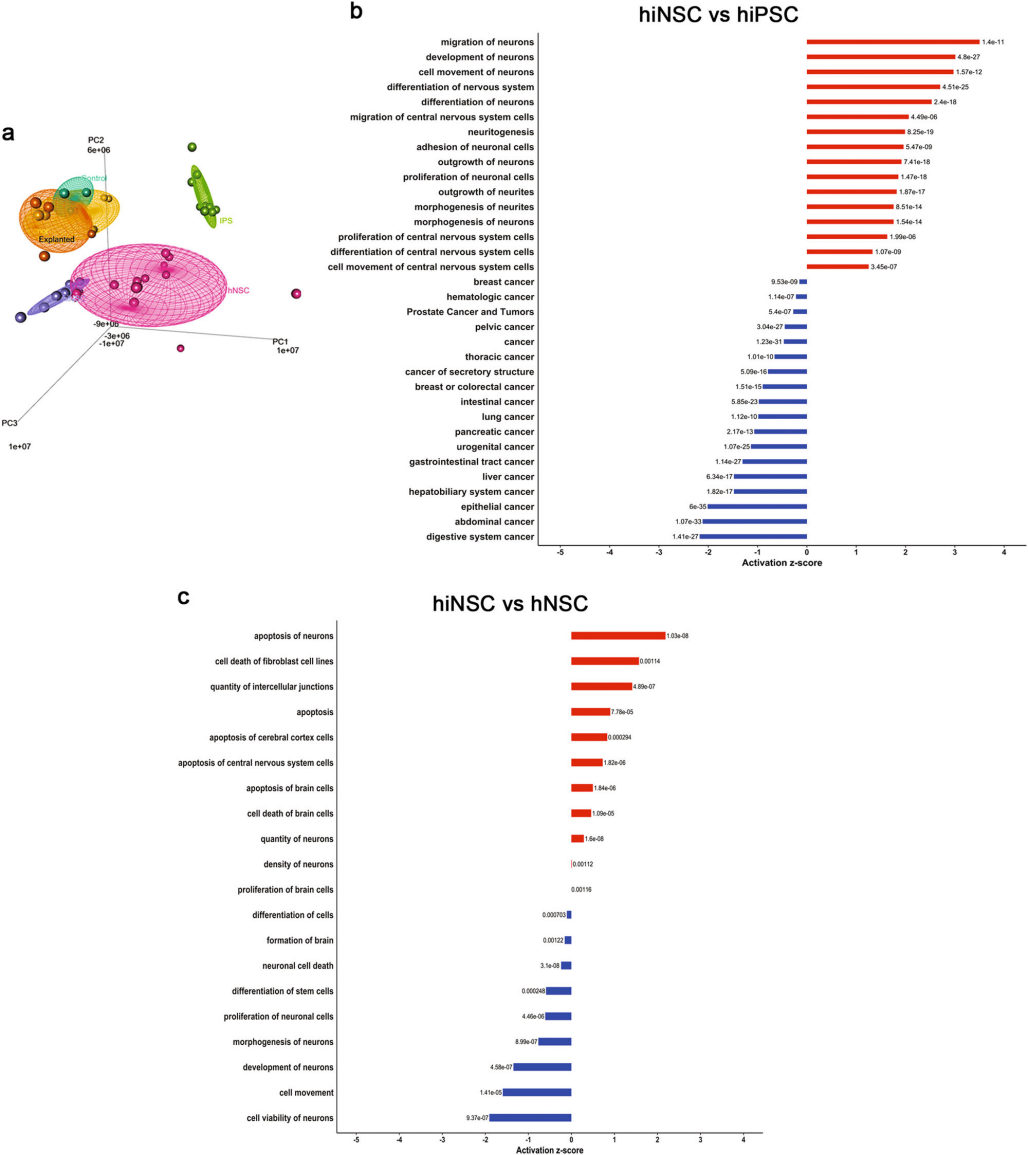
Gene expression analysis. **a** Principal component analysis showing the whole-genome expression profiles of hiNSCs (purple cloud) in comparison with: hiPSCs (light green cloud, including hiPSC#1, hiPSC#2, hiPSC#3 and IPS lines derived from the public database GSE61358), hNSCs (pink cloud, including the GMP-grade foetal hNSC line described in this article and data derived from the public database GSE61358) and three glioblastoma cell lines (yellow, orange and dark green clouds, obtained from the public database GSE72218). **b** Differential gene expression of hiNSCs versus their parental hiPSCs. **c** Differential gene expression of hiNSCs versus foetal hNSCs

We analysed the whole-genome expression profiles of hiNSCs compared to: (1) parental hiPSCs and IPS lines derived from public database GSE61358^42^; (2) the hNSCs described in this article and NSCs derived from public database GSE61358; and (3) three glioblastoma cell lines obtained from public database GSE72218. Principal component analysis (PCA) (Fig. 6a) showed that the cell lines clustered together according to cell type, demonstrating that the inter-type cell line differences were maintained with respect to their genetic background. hiNSCs clustered closest to hNSCs, while undifferentiated iPS formed a separate cluster, which was closer to the group of glioblastoma cell lines. The comparison between hiNSCs and parental hiPSCs showed that the genes specifically expressed by hiNSCs were related to movement (*z*-score = 1.702, *p* < 0.0001), cell proliferation (*z*-score = 0.833, *p* < 0.0001) and differentiation of CNS cells (*z*-score = 1.186, *p* < 0.0001), while genes implicated in cancer (typical of hiPSCs) were silenced (Fig. 6b). These results supported our “in vitro” and “in vivo” experiments and confirmed the non-tumorigenic properties of these cells. Moreover, differentially expressed genes in the hiNSC group were shown to be significantly implicated in the process of apoptosis of brain cells (*z*-score = 0.770, *p* = 0.0023) and quantity of neurons (*z*-score = 0.901, *p* < 0.0001), which were turned on in these cells, while genes implicated in cell proliferation were turned off, as opposed to the hNSC group (Fig. 6c). This result is in line with our experimental data that demonstrated that hiNSCs have a lower growth rate than hNSCs.

## Discussion

Extensive preclinical studies have furthered the use of neural precursors in clinical trials^4,35,43–46^. Furthermore, over 100 patients have now received implantations of hNSCs in authorised clinical trials worldwide^14–18,47–49^.

Extending these initial approaches to a larger body of patients is critically dependent on the availability of suitable sources of donor cells, which must retain reproducible and predictable cellular characteristics over a timespan long enough to generate an adequate amount of cells. This guarantees both that the treatment will be available to many patients and that all of these will receive the same cellular therapeutic agent for as long as necessary.

This manuscript describes a protocol for establishing hiPSC-derived hiNSCs, whose virus-free generation and stem cell characteristics provide an ideal source of autologous brain cells amenable for cell therapies in humans, showing that it is possible to isolate cells possessing all expected characteristics of bona fide hNSCs from skin fibroblasts^50–52^.

Generating hiNSCs from hiPSCs as described here may have several significant advantages. First, this system allows for two sequential amplification steps, the first regarding hiPSC growth and the second at the stage of hiNSCs, rendering the number of cells that can be used in neurological disorders virtually unlimited. Second, genetic manipulation can be carried out at the hiPSCs stage, passed on to the hiNSCs, which could be transplanted into patients. Third, the generation of autologous hiNSCs circumvents the need for immunosuppression regimens. Finally, on the basis of the functional homology between hiNSCs and clinical-grade hNSCs, our hiNSCs will facilitate cGMP certification previously granted to hNSCs.

Like their foetal hNSC counterparts, our hiNSCs had a stable expansion rate, did not possess telomerase activity, remained strictly dependent on mitogens for their proliferation and expansion and when growth factors were withdrawn, promptly differentiated into neurons, astroglia and oligodendrocytes. Of note, hiNSC correctly executed the differentiation process, as it normally occurs in hNSCs, so that the appropriate, univocal segregation of single pan-lineage-specific markers occurred within distinct, single differentiated cells (Fig. 3c), as opposed to the promiscuous expression observed in GBM cells^41^. Along the initial 24 days of in vitro differentiation, the number of hiNSC-derived neurons increased linearly over time (as for hNSCs), with a reduction on the 31st DIV. Interestingly, in the mouse brain, where trophic factors were present, at 6 months from transplantation, the percentage of hiNSC-derived neurons was greater with respect to hNSCs. It is likely that the in vitro experimental conditions are not optimal for full survival after differentiation.

Previous approaches have so far employed morphogenetic molecules to induce iPSC neuralisation^53–57^. We hypothesised that during long-term hiPSC-derived EB differentiation, radial glial cells, which are responsible for adult neurogenesis in the subventricular zone of the mammalian brain^58,59^, would develop. Having observed that this did indeed occur within a subset of cells, we applied a positive cell selection through a chemically defined medium. This permitted the exclusive expansion of the hiNSCs without the further addition of fate specification molecules. Significantly, our cells do not need to be committed towards specific neural differentiation before being inserted into the human brain, unlike formerly published protocols^60–62^, but could in the future be applied as single undifferentiated hiNSCs, whose differentiation would occur spontaneously through stimulation by the natural environment in which they will have been transplanted.

Our hiNSC preparation technique requires 2 months, followed by 4–8 months for line amplification, a much longer time window than those previously described^54–57^. However, these protocols do not stably amplify hNSCs as derived from GMP-grade iPCs. Thus, the dual advantages of our protocol are that we can produce these cells without adding inducing molecules and that neural cells can be obtained in the large amounts required for several serial transplants.

During our procedure, we repeatedly encountered two different types of cells, which appeared morphologically and functionally identical up to the sixth passage in culture, when differences emerged: the first type fit the definition of Transit Amplifying Progenitor (TAPs), which after a short proliferation (maximum seven passages) stopped growing and subsequently died (Suppl. Figure 6d). The second type fit the definition of bona fide hNSCs, corresponding to our hiNSCs, which continued to grow and thrive up through 25 passages. This has been explained in the literature^58,59,63^, which underlines the significant differences between these two groups of precursors: (i) bona fide stem cells, whose self-renewal spans or even exceeds that of the organism and (ii) short-term self-renewing TAPs, which have an intrinsically restricted self-renewal ability and terminally differentiate after a limited number of cycles^43,44^. These two types of cells possess very different functional and molecular properties, which have relevant consequences on their potential therapeutic applications. hNSC in general, and the hiNSCs described here, will sustain a long-term stable expansion process suitable for certifiable clinical-grade cell drug products with standard properties, TAPs will not. Rigorous long-term experiments should be performed on NSC lines, as mistaking TAPs for hNSCs may engender confusion and create significant issues with standardisation and reproducibility of results in cell therapy.

We further confirmed the safety of our hiNSCs and their similarity to cGMP-grade hNSCs, observing that transplantation into the striatum of athymic nude mice (6 months) showed no clinical symptoms known to accompany cell overgrowth in the brain. Histological analyses confirmed that none of our hiNSCs established hyperplastic or neoplastic masses or formed the typical teratomas observed after implantation of their parental iPSCs. Corroborating the results obtained in animal models, the first patient treated with cGMP hNSCs, has had a follow-up of more than 6 years without evidences of abnormal cell proliferation^64,65^.

Future studies will allow us to expand this approach and to define the full spectrum of possibilities that our technique offers for producing different subtypes of hiNSCs and for obtaining autologous brain stem cells that are amenable for therapy in humans. In reference to these objectives, hiNSCs produced with our protocol are not only suitable for receiving certification for safe implantation into humans, but may contribute to resolving many of the practical, regulatory and immunological issues that still afflict the use of foetal hNSCs in clinical settings.

## Materials and methods

### Production and characterisation of hiPSC lines

The hiPSCs used in this study were iPS#1, iPS#2 and iPS#3. The first two lines were from a 41-year-old female donor and the third from a female donor aged 51. The lines were established through virus-free reprogramming. Briefly, we nucleofected 3 × 10^5^ fibroblasts with 3 μg of a combination of three episomal vectors (pCXLE-hOCT3/4-shp53—Addgene #27077, pCXLE-hSK—Addgene #27078 and pCXLE-hUL—Addgene #27080). Six days after nucleofection, cells were detached and re-seeded onto a matrigel layer (Corning) and cultured in NutristemXF medium (Biological Industries). Small hiPSC colonies became visible between 5 and 6 weeks after transfection. We obtained a reprogramming efficiency of about 0.005% and 0.004% respectively for patient 1 and patient 2, corresponding to 16 and 12 iPSC clones. Each hiPSC line exhibited typical hESC morphology and expressed standard pluripotency markers, detected through immunocytochemistry (anti-OCT4 (1:100—Life technologies) and anti-TRA-1-60 (1:100—Life Technologies)) and qRT-PCR (for primers list see Supplementary table 1). The hiPSC colonies were mechanically detached and amplified once a week. If spontaneously differentiated colonies appeared, manual removal was carried out. We also tested their potentiality to differentiate into three embryonic layers, both through EB and teratoma formation assays. For the generation of EBs, cells were re-suspended in Dulbecco’s modified Eagle’s medium (DMEM)/F12 medium supplemented with 20% KSR, 0.1 mM non essential amino acids (NEAA), 1 mM l-glutamine, 50 μM 2-mercaptoethanol, 50 U/mL penicillin and 50 mg/mL streptomycin. Fourteen days later, EBs were pelletted and RNAs were extracted for qRT-PCR analysis. For teratoma formation, approximately 3 × 10^6^ dispase-treated hiPSCs, in 100 μL of Matrigel, were injected into the right flank of nude mice. After 1 month, tumours were collected for histological analysis to check for their in vivo differentiation capacity into derivatives of all three germ layers. Teratomas were included in paraffin; standard haematoxylin-eosin and Alcian Blue staining were performed. For immunohistochemistry the following antibodies were used: VIMENTIN (DAKO, M0725), S100 (DAKO, Z0311), biotinylated goat anti-rabbit (DAKO, E0432) and biotinylated goat anti-mouse (DAKO, E0433). Absence of mycoplasma contamination was verified by PCR analysis using N-Garde Mycoplasma PCR kit (EuroClone). For karyotyping, iPSCs were cultured in chamber slides (Thermo Fisher Scientific) coated with Matrigel (1:100) in Nutristem medium for 2–3 days. Cells were treated with a COLCEMID solution (Thermo Fisher Scientific) with a final dilution of 0.1 µg/mL for 60 min at 37 °C. Metaphases were obtained by adding a hypotonic solution (30 mM KCl in 10% foetal bovine serum (FBS)), followed by incubation at 37 °C for 6 min and by fixation using a cold, freshly made 3:1 ethanol:acetic acid solution. Karyotype analysis was carried out on GTG-banded metaphases (resolution 450–500). Fifteen metaphases were counted and three karyotypes analysed. Only clonal aberrations were considered: an identical structural alteration or the gain or loss of a chromosome had to appear contemporarily in at least two or three different cell colonies, as specified in international system of human cytogenetic nomenclature (ISCN) recommendations.

### Derivation and maintenance of human NSC lines

Each hiPSC line was expanded to at least 70% confluency. Spontaneously differentiated colonies were manually removed via gentle scraping and hiPSCs were then detached using 1 mg/mL dispase (Sigma) in DMEM-F/12. After gentle scraping, cell aggregates were placed into Petri plates and cultured as cell suspension in NutristemXF medium. This medium was substituted after 3 days with KSR medium (DMEM/F12, 20% Knock-out Serum Replacement (Life Technologies), 2 mM l-glutamine, 0.1 mM beta-mercaptoethanol and 1% non-essential amino acids). We maintained the embryoid bodies for 53 days in KSR medium, putting them in hypoxic conditions (5% CO_2_ and 5% O_2_) after the first 2 weeks. The medium was changed twice a week. After 53 days we began the selection of neural precursor cells with serum-free neurosphere growth medium^8^. After 10 days, cells were collected, mechanically dissociated into single cells and replated at high density in the same medium. In this phase, a single cell produces a sphere that can be routinely split every 10–15 days: up to 20–30 times. The mycoplasma contamination test and karyotype analysis were performed with the same protocols as those used for iPS cells; the only difference, for karyotype analysis, was the plating of neurosphere cells, which were attached onto cultrex and grown in the neurosphere medium to amplify cells. CNV analysis was performed using an SNP-array platform (Cytoscan HD; Affymetrix, Santa Clara, CA), following the manufacturer’s instructions. Labelled DNA was hybridised for 16–18 h at 49 °C in a GeneChip Hybridisation Oven 645 (Affymetrix). The chip was washed, stained in the GeneChip Fluidic Station 450, and scanned with a 3000 7 G (Affymetrix) scanner. Copy number analysis was performed using Affymetrix Chromosome Analysis Suite software (ChAS v3.0; Affymetrix). CNVs were filtered as follows: 25 markers and 500 kb of minimal size.

### hiNSC cryopreservation protocol

Neurospheres were collected in a 15 mL tube and pelleted by centrifugation. The supernatant was discarded, the pellet was gently re-suspended in 1.5 mL of growth medium supplemented with 10% dimethylsulphoxide and transferred to a cryovial. The cryovial was placed into a freezing jar containing isopropyl alcohol, kept for at least 4 h at −80 °C and finally transferred to the liquid nitrogen tank.

To thaw hiNSCs, the cryovial was transferred in a 37 °C bath until thawed. Cells were transferred into a 15 mL tube containing 5 mL of growing medium and pelleted by centrifugation. The supernatant was discarded and cells gently re-suspended in growing medium and transferred to a cell culture flask to allow for further expansion^36^. Vitality of thawed cells was evaluated at the first amplification passage by counting viable cells out of the total cell number.

### Neural differentiation

Neurospheres were mechanically dissociated to yield a single-cell suspension and transferred onto cultrex-coated glass coverslips at the density of 10 000 cells/coverslip in neurosphere growth medium consisting exclusively of fibroblast growth factor 2 (20 ng/mL). Cultures were shifted after 72 h to a mitogen-free medium containing 2% FBS (default differentiation protocol). Differentiated cells were cultured for up to 5 weeks to obtain a mixture of neural cells containing astrocytes, neurons and oligodendrocytes. Immunofluorescence analysis was performed at different stages of differentiation: 10, 17, 24 and 31 days after plating (see below).

The spontaneous differentiation of hiNSCs in our protocol was used exclusively to demonstrate their multipotency; in fact, the neurons and astrocytes were not intended for subsequent utilisation in humans.

### Immunocytochemistry and immunohistochemistry

Cultures were fixed for 10 min in freshly buffered 4% paraformaldehyde at room temperature, followed by two 1× PBS washes. After blocking with 10% normal goat serum (NGS), the cultures were incubated overnight at 4 °C with the following antibodies: anti-TUBB3 (1:400—BioLegend); GFAP (1:200—Dako); GALC (1:200—Merk Millipore); anti-GLU; and anti-GABA. After rinsing with PBS, cultures were incubated with the following secondary antibodies: anti-rabbit Alexa Fluor 488 and anti-mouse Alexa Fluor 555 (Invitrogen). Cultures were then stained with Hoecht 33342 (Invitrogen) or 49,6-diamidino-2-phenylindole (DAPI) for nuclear staining. Microphotographs were taken, using a Nikon C2 fluorescence microscope and NIS Elements 1.49 software. Data are reported as percentages of labelled cells over the total number of nuclei ± SEM. Each value represents the average of at least three independent experiments.

### RT and qRT-PCR

Total RNAs were isolated from fibroblasts, hiPSCs and hiNSCs of each patient using TRIzol reagent (Life Technologies), following the manufacturer’s instructions. RNA quality was assessed by determining ultraviolet 260/280 absorbance ratios at Nanodrop 1000 (Thermo Scientific) and examining RNA size distribution on RNA 6000 Nano LabChips (Agilent Technologies), processed on the Agilent 2100 Bioanalyzer, using the total RNA electrophoresis programme. Only RNAs with a RNA integrity number ≥ 8 were used for subsequent analysis.

Reverse transcription was performed using a High Capacity cDNA Reverse Transcription Kit (Applied Biosystems), following the manufacturer’s instructions, after digestion with DNAse I (Life Technologies).

qRT-PCR was performed using a 7900HT Fast Real-Time PCR system (Applied Biosystem). For each gene of interest, qRT-PCR was performed as follows: each RNA sample was tested in duplicate and B-ACTIN was used to normalise transcript abundance and calculations were performed with the 2^DeltaDeltaCt method. Statistical analyses were performed on at least three independent experiments.

Sybr green reactions were performed using Power SYBR Green PCR Master Mix (Applied Biosystem) with the following PCR programme: denaturation 95 °C for 10 min; amplification 95 °C for 10 s, 60 °C for 10 s, 72 °C for 30 s, all of which was repeated for 50 cycles; final elongation 72 °C for 7 min; final dissociation step 95 °C for 15 s, 60 °C for 15 s and 95 °C for 15 s. TaqMan reactions were carried out using TaqMan Universal PCR Master Mix (Applied Biosystem) and PCRs were performed, following the manufacturer’s instructions.

Primers are listed in Supplementary Table 1.

### Animal studies

Animal studies were approved by the Italian Ministry of Health Ethics Review Committee for Animal Experimentation, following protocol AUT. 651/2016 PR. Adult female Hsd Athymic Nude-Foxn1^nu^ (Envigo) (15–20 g). hiNSCs were seeded (1 × 10^5^ cells/cm^2^) in growth medium for 24 h. On the day of transplantation, cells were counted and re-suspended in Hank’s balanced salt solution (medical) (density of 1 × 10^5^ cells/μL). Mice striatum were unilaterally and stereotaxically (David Kopf Instruments, Tujunga, CA) injected with 3 μL of each cell suspension: hiNSCs and brain-derived hNSCs, 3 × 10^5^ cells/mouse, *n* = 21; GBM cells: 150 000 cells/3 μL/animal, *n* = 3. Animals were analysed for 6 months (for hNSCs) and 3 months (for GBM). For the immunohistochemical analysis, mice were euthanized and transcardially perfused-fixed with 4% paraformaldehyde. Brains were post-fixed overnight, cryoprotected, frozen and coronally sectioned (20-μm thick) by cryostat. Sections were blocked with 10% NGS and 0.3% Triton X-100 for 90 min. Primary antibodies used: huN, NCL-KI67p (KI67, Novocastra), human NESTIN (NES) (R&D Systems, Minneapolis), GFAP (Dako Cytomation), TUBB3, MBP and DCX (Santa Cruz). Fluorescent secondary antibodies were labelled with Alexa Fluor 549 and 488 (Molecular Probes). DAPI (ROCHE) or TO-PRO3 Iodide (Molecular Probes) were used as nuclear markers. Labelled samples were analysed by fluorescence microscopy (Zeiss Axioplan 2 imaging) and by confocal microscopy (Leica DM IRE2). The survival rate of transplanted cells was evaluated by counting huN+ cells in serial brain sections (each 20 μm apart) spanning the graft area. The total number of surviving transplanted cells was calculated for the whole graft using the Abercrombie formula (Abercrombie, 1946). Data are presented as the average percentage of surviving cells over total transplanted cells (300 000). The antero-posterior migration was calculated by evaluating the distance between the most proximal and most caudal section containing huN+ cells. The evaluation of proliferating cells and neural phenotypes derived from transplanted cells was performed by calculating the percentage of huN+ cells co-expressing, respectively, Ki67, NES, GFAP, TUBB3, MBP and DCX out of the total huN+ cells in three serial sections of the transplanted animals (*n* = 8).

### Bioinformatics analysis

The whole-transcriptome profiles of the herein described hiPSCs, hiNSCs and hNSCs were compared with those of publicly available data sets (GSE72218^42^ and GSE61358). These Gene Expression Omnibus data sets belong to the platform GPL17586, which contains data generated with Affymetrix Human Transcriptome Array 2.0 technology. In particular, in GSE72218, total RNA was extracted from both tumour tissues and three cell lines (U3020MG, U3047MG and U3065MG) intracranially transplanted into nonobese diabetic/severe combined immunodeficiency mice. The second cohort, GSE61358, concerns total RNAs extracted from hiPSCs and induced pluripotent stem cell-derived NSCs (iPS-derived neural precursor cell NPC). Our raw data, together with those of GSE72218 and GSE61358, were analysed through the Exploratory Grouping Analysis (EGA) available from the Transcriptome Analysis Console (TAC) 4.0 software. The first three principal components (PC) of the PCA of EGA were visualised through the rgl package of R software (ver 3.4.4). Differential expression analysis was performed using TAC (one-way analysis of variance (ANOVA)) and only genes with differential expression greater (absolute values) than twofolds were taken into consideration for further analyses. Functional enrichment analysis was performed by Ingenuity Pathway Analysis (IPA^®^) software (QIAGEN Inc.).

### Statistical analysis

One-way ANOVA and Student’s *t*-test were performed using Excel programme. *p* Values < 0.05 were considered statistically significant. Results are presented as means ± SEM. *p < 0.05; **p < 0.01; ***p < 0.001.

## Electronic supplementary material

Suppl.Figure 1

Suppl.Figure 2

Suppl.Figure 3

Suppl.Figure 4

Suppl.Figure 5

Suppl.Figure 6

Table 1

supplementary figure legends
