# Supplementary figures and images for: Establishment of stable iPS-derived human neural stem cell lines suitable for cell therapies
Source: Cell Death Dis. 2018 Sep 17;9(10):937. doi: 10.1038/s41419-018-0990-2 (PMC6141489; doi:10.1038/s41419-018-0990-2)

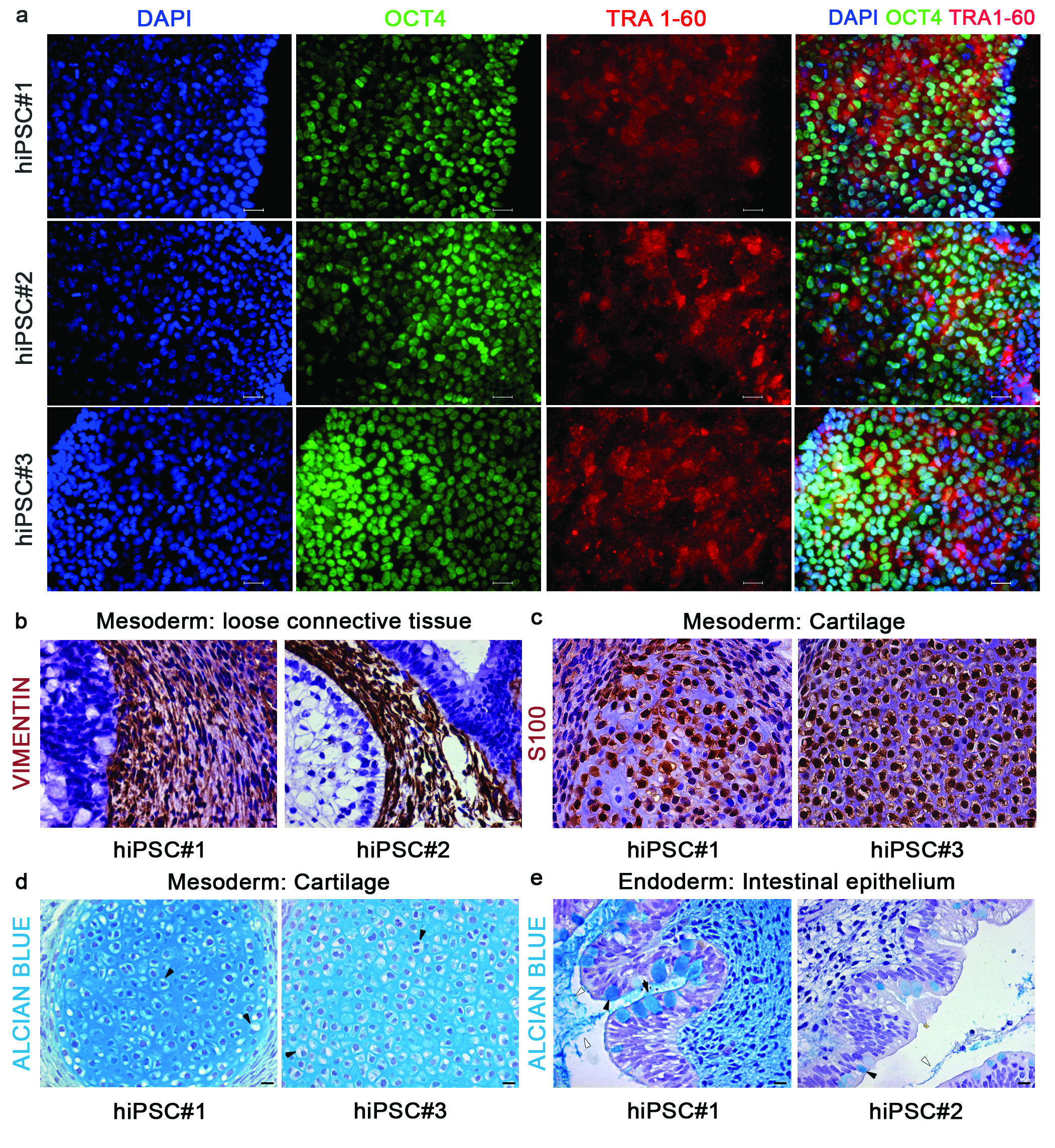

Supplement: Supplementary file 1 — Suppl.Figure 1 [file 41419_2018_990_MOESM1_ESM.tif]

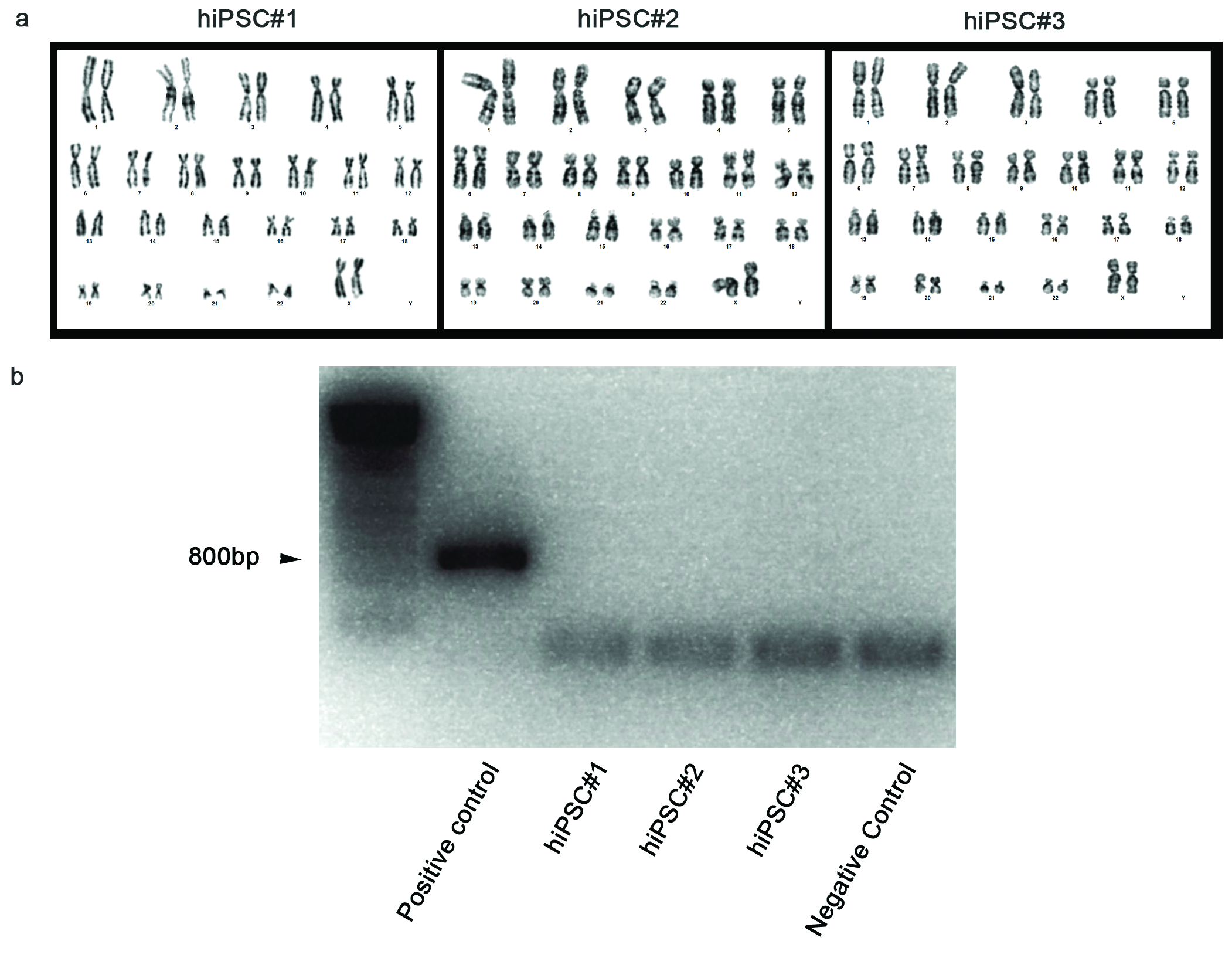

Supplement: Supplementary file 2 — Suppl.Figure 2 [file 41419_2018_990_MOESM2_ESM.tif]

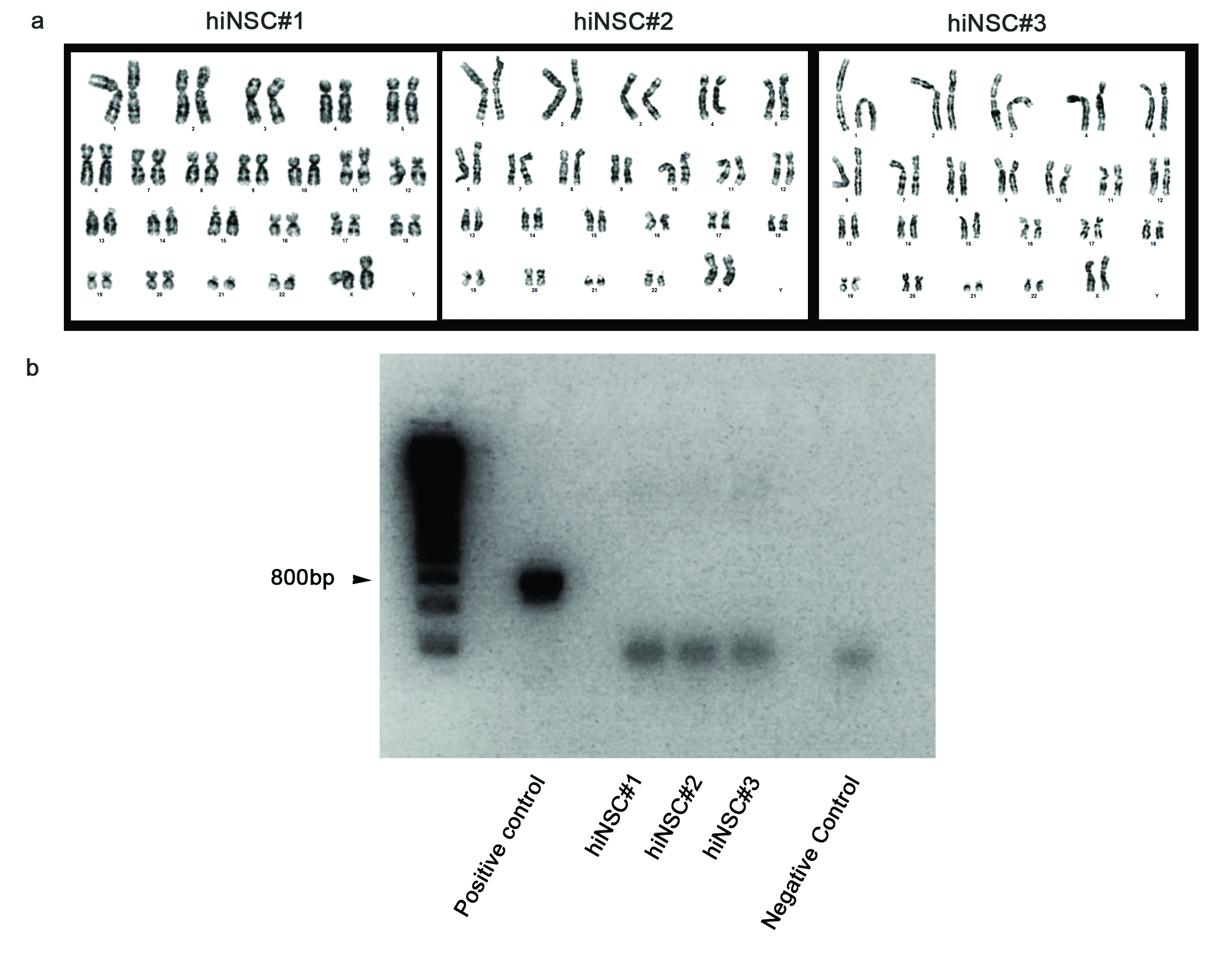

Supplement: Supplementary file 3 — Suppl.Figure 3 [file 41419_2018_990_MOESM3_ESM.tif]

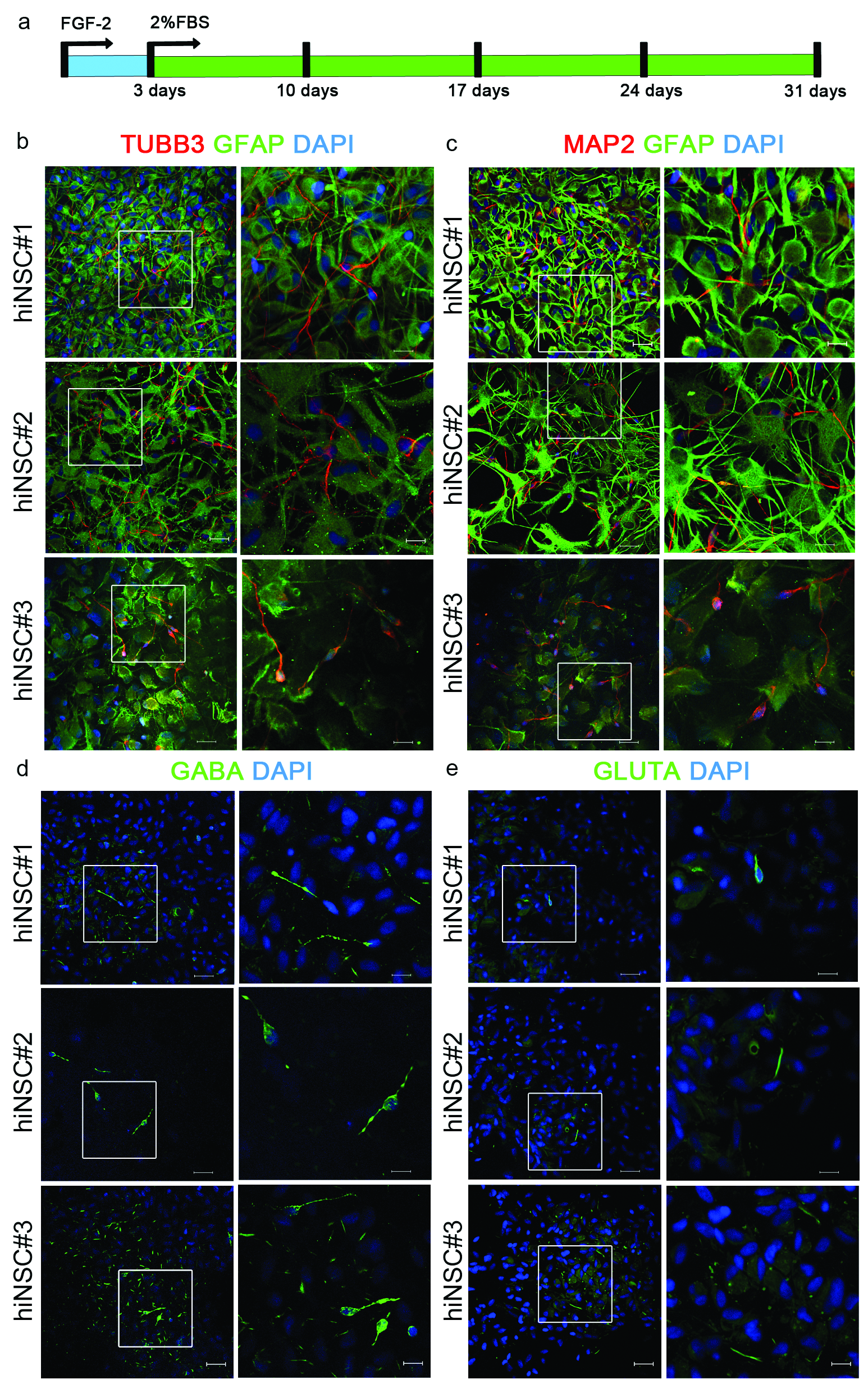

Supplement: Supplementary file 4 — Suppl.Figure 4 [file 41419_2018_990_MOESM4_ESM.tif]

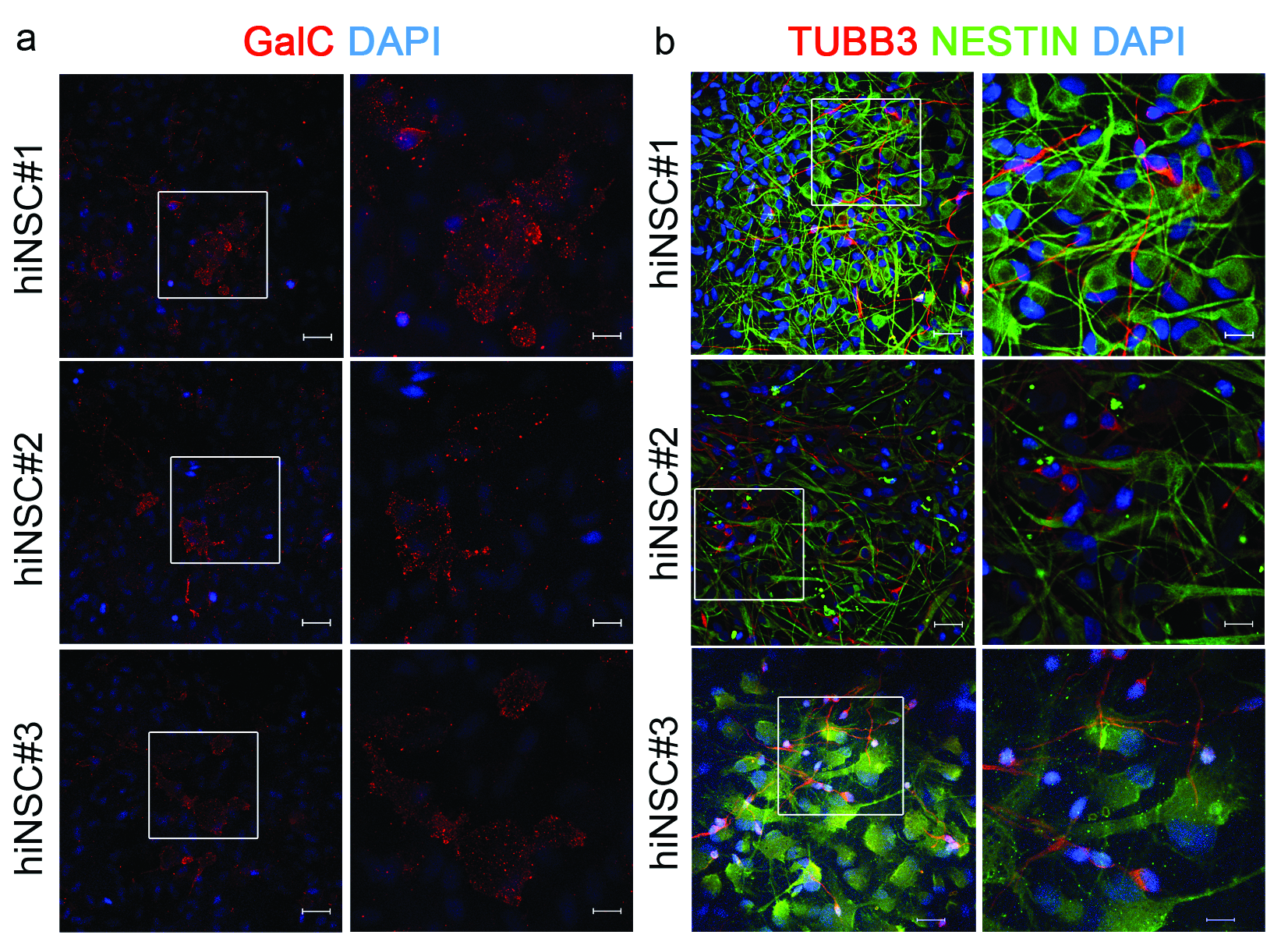

Supplement: Supplementary file 5 — Suppl.Figure 5 [file 41419_2018_990_MOESM5_ESM.tif]

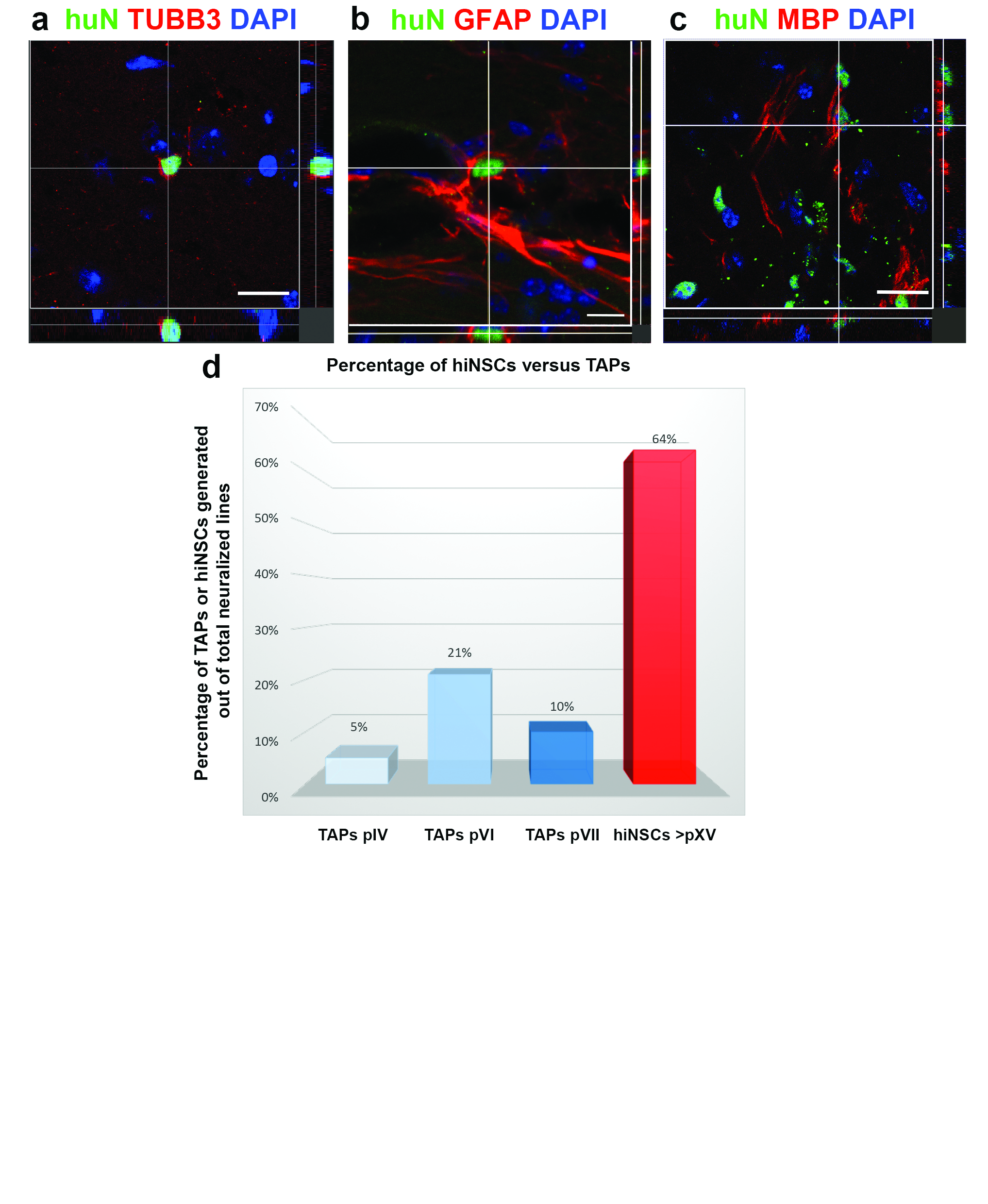

Supplement: Supplementary file 6 — Suppl.Figure 6 [file 41419_2018_990_MOESM6_ESM.tif]
