# Supplementary material for: Establishment of stable iPS-derived human neural stem cell lines suitable for cell therapies
Source: Cell Death Dis. 2018 Sep 17;9(10):937. doi: 10.1038/s41419-018-0990-2 (PMC6141489; doi:10.1038/s41419-018-0990-2)
Supplement: Supplementary file 7 — Table 1 [file 41419_2018_990_MOESM7_ESM.pdf]

Primers		
	**Target**	**Forward/Reverse primer (5′-3′)**
Episomal genes	*eOCT4*<br><br><br>*eKLF4*<br><br><br>*eLIN28*<br><br><br><br>*eL-MYC*<br><br><br><br>*eSOX2*	*Fwd: CAT TCA AAC TGA GGT AAG GG*<br>*Rev: TAG CGT AAA AGG AGC AAC ATA G*<br><br>*Fwd: CCA CCT CGC CTT ACA CAT GAA GA*<br>*Rev: TAG CGT AAA AGG AGC AAC ATA G*<br><br>*Fwd: AGC CAT ATG GTA GCC TCA TGT CCG C*<br>*Rev: TAG CGT AAA AGG AGC AAC ATA G*<br><br>*Fwd: GGC TGA GAA GAG GAT GGC TAC*<br>*Rev: TTT GTT TGA CAG GAG CGA CAA T*<br><br><br>*Fwd: TTC ACA TGT CCC AGC ACT ACC AGA*<br>*Rev: TTT GTT TGA CAG GAG CGA CAA T*
Pluripotency genes	*OCT4*<br><br><br><br>*KLF4*<br><br><br><br><br>*LIN28*<br><br><br><br><br>*L-MYC*<br><br>*SOX2*	*Fwd: CCC CAG GGC CCC ATT TTG GTA CC*<br>*Rev: ACC TCA GTT TGA ATG CAT GGG AGA GC*<br><br><br>*Fwd: ACC CAT CCT TCC TGC CCG ATC AGA*<br>*Rev: TTG GTA ATG GAG CGG CGG GAC TTG*<br><br><br><br>*Fwd: AGC CAT ATG GTA GCC TCA TGT CCG C*<br>*Rev: TCA ATT CTG TGC CTC CGG GAG CAG GGT AGG*<br><br><br><br>*Fwd: GCG AAC CCA AGA CCC AGG CCT GCT CC*<br>*Rev: CAG GGG GTC TGC TCG CAC CGT GAT G*<br>*Fwd: TTC ACA TGT CCC AGC ACT ACC AGA*<br>*Rev: TCA CAT GTG TGA GAG GGG CAG TGT GC*
House-Keeping Genes	*18S*	*Fwd: GGC CCT GTA ATT GGA ATG AGT C*<br>*Rev: CCA AGA TCC AAC TAC GAG CTT*
Differentiation Markers	*NESTIN*<br><br>*PAX6*<br><br>*EOMES*<br><br>*T*<br><br>*GATA4*	*Hs04187831_g1*<br><br>*Hs00240871_m1*<br><br>*Hs00172872_m1*<br><br>*Hs00610080_m1*

	FOXA2	*Hs00171403_m1*
	18S	*Hs00232764_m1*
		Hs03003631_g1

Table 1
