# Supplementary material for: Establishment of stable iPS-derived human neural stem cell lines suitable for cell therapies
Source: Cell Death Dis. 2018 Sep 17;9(10):937. doi: 10.1038/s41419-018-0990-2 (PMC6141489; doi:10.1038/s41419-018-0990-2)
Supplement: Supplementary file 8 — supplementary figure legends [file 41419_2018_990_MOESM8_ESM.docx]

**Supplementary Figure Legends**

**Suppl. Figure 1. Characterization of adult skin fibroblasts-derived hiPSC lines.** (a) Immunofluorescence images showing expression of OCT4 (green) and TRA1-60 (red) in hiPSCs lines. Nuclei are counterstained with DAPI (blue). (b) Bright field images showing VIMENTIN-positive loose connective tissue generated by the differentiation of hiPSC#1 and hiPSC#2 within subcutaneous implants. Interestingly, within teratomas derived from hiPSC#2, the mesodermal tissue was located amongst ectodermal (left) and endodermal (right) structures, that do not express VIMENTIN. Thus recapitulating the three-layered embryo. (c-d) Primitive cartilage formation composed by clusters of chondroblasts (expressing S100, c) embedded in the characteristic proteoglycans rich-cartilaginous matrix (blue stained in d). More mature chondrocytes, included in the typical lacunae, are shown in d (black arrowheads). (e) Characteristic endodermal epithelium containing mucin-producing goblet cells (black arrowheads in e). The distinctive mucoid secretions (blue stained), is evident both within the cells (black arrowheads in e) and within the lumen bounded by the primitive intestine-like villi (white arrowheads in e), consistently with the glandular phenotype of the cells. Scale Bars: in (a) = 20 μm in (b-e) = 10 μm

**Suppl. Figure 2. Characterization of adult skin fibroblasts-derived hiPSC lines.** (a) Karyotype of hiPSC#1, hiPSC#2, hiPSC#3. No evident chromosome alterations were present in hiPSCs (b) PCR showing that hiPSCs cultures were mycoplasma free.

**Suppl. Figure 3. Characterization of hiNSCs** (a) Karyotype of hiNSC#1, hiNSC#2, hiNSC#3. No evident chromosome alterations were present in hiNSC lines (b) PCR showing that hiNSCs cultures were mycoplasma free.

**Suppl. Figure 4. Multipotency of hiNSCs.** (a) Schematic of the differentiation protocol applied to asses multipotency of hiNSC lines. Cells were plated in adhesion onto coultrex and allowed to differentiate in growing medium without EGF. Three days after, the medium was replaced with one deprived of growth factors with the addition of 2% fetal bovine serum (FBS) (b-c). Representative confocal images showing hiNSC-derived neurons TUBB3 positive, (red, b) and MAP2 positive (red, c), astrocytes (GFAP positive, green, b and c) at days 24 upon differentiation. In the right panel is shown a magnification of the boxed area outlined in the left panel. (d-e) Expression of (d) GABA (green) (e) GLUTA (green) in hiNSCs, after 24 days of *in vitro* differentiation. In the right panel is shown a magnification of the boxed area outlined in the left panel. Scale bars: (a-d) left panel =20 μm; (a-d), right panel = 10 μm.

**Suppl. Figure 5. Multipotency of hiNSCs.** (a) Representative confocal images showing hiNSC-derived oligodendrocytes (GalC, red) at days 24 upon differentiation. In the right panel is shown a magnification of the boxed area outlined in the left panel. (b) Representative confocal images showing hiNSC-derived neurons TUBB3 positive, (red) and NESTIN expression (green) at days 24 upon differentiation. In the right panel is shown a magnification of the boxed area outlined in the left panel. Scale bars: (a,b) left panel =20 μm; (a,b), right panel = 10 μm.

**Suppl. Figure 6. Differentiation of hiNSCs upon transplantation into the brain and TAPs occurrence.** Representative confocal images showing the co-localization of the neuronal markers TUBB3 (a, red), the astroglial marker GFAP (b, red) and the oligodendrocyte marker MBP (c, red) with huN (a-c, green) that identifies hiNSCs nuclei with at 6 months upon transplantation. (d) Chart showing the occurrence of TAPs production along the neuralization process. In the chart is shown the percentage of lines that did not grow beyond sixth-seventh amplification passages (therefore considered TAPs) and lines that were expanded for more than fifteenth passages (considered *bona fide* hNSC lines). Scale bars: in (a) 17,7 μm; in (b) = 10 μm; in (c) = 16,15 μm.
